# Correction: Confidence interval estimation of the common mean of several gamma populations

**DOI:** 10.1371/journal.pone.0294204

**Published:** 2023-11-03

**Authors:** Li Yan

There are errors in the Funding section. The correct Funding statement is: This work was supported by National Cancer Institute (NCI) grant P30CA016056 involving the use of Roswell Park Comprehensive Cancer Center’s Pathology Network, Genomics and Biomedical Data Science Shared Resources.
