# Supplementary material for: Confidence interval estimation of the common mean of several gamma populations
Source: PLoS One. 2022 Jun 17;17(6):e0269971. doi: 10.1371/journal.pone.0269971 (PMC9205481; doi:10.1371/journal.pone.0269971)
Supplement: Attachment — Submitted filename: Report.pdf [file pone.0269971.s001.pdf]

Comments on the paper "Confidence interval estimation of the common mean of several gamma populations"

The author has addressed the problem of estimating the common mean of several gamma distributions. He has proposed several confidence intervals (CIs) based on the generalized variable approach, MOVER and likelihood approach. The proposed CIs are evaluated and compared with respect to coverage probabilities and expected widths. In general, the paper is easy to read and I have the following comments and suggestions.

1. The authors should provide some justifications for the large sample CI in (4).
2. For easy reference, it is better to give the PDF of the two-parameter gamma distribution.
3. Page 2, first line: We will first present....
4. Page 3: The U_1 and U_2 in R_ν and R_{σ^2} are the same.
5. There is a typo in (9).
6. The line below (9): What is $\hat{\alpha}^*$? Is it the MLE?
7. Among all CIs, the one with good coverage probabilities and smaller expected widths is preferable to others. So it is better to present the coverage probabilities along with expected widths in the same table. Alternatively, compare the CIs with respect to coverage probabilities and select those with good/satisfactory coverage properties, and compare only those with respect to expected widths.
