# Supplementary material for: Confidence interval estimation of the common mean of several gamma populations
Source: PLoS One. 2022 Jun 17;17(6):e0269971. doi: 10.1371/journal.pone.0269971 (PMC9205481; doi:10.1371/journal.pone.0269971)
Supplement: Attachment — Submitted filename: Responses_to_reviewer_2.pdf [file pone.0269971.s002.pdf]

Response to Reviewer 2 (PONE-D-21-27089): "Confidence Interval Estimation of the Common Mean of Several Gamma Populations" by Yan

We appreciate your reading and valuable comments/suggestions which greatly improved this article. For your consideration, the following point-to-point responses are provided.

1. *What is the rationale behind the constructed GPQs (11) and (12)? Are they valid GPQs? Are there any theoretical guarantees of using them in constructing the confidence intervals?*

Response:

Thanks for the questions.

Eq (11) is simply a result of standard substitution methods; e.g. $R_{var(\hat{\mu}_i)}$, i.e. the generalized pivot of $var(\hat{\mu}_i)$ is obtained by substituting μ_i and α_i using their corresponding pivots R_{μ_i} and R_{α_i} in eq (2) , i.e. $var(\hat{\mu}_i) = \frac{\mu_i^2}{n_i\alpha_i}$. This method is routinely used in fiducial inference (e.g. Hannig et al. JASA, 2006) for constructing pivots.

Eq (12) leads to bona fide generalized pivot. This way of constructing generalized pivots for common mean has been widely used in literature under a variety of settings. For example, Krishnamoorthy and Lu (Biometrics, 2003) studied inferences on the common mean of several normal populations based on the generalized variable method; Tian and Wu (Biometrical

journal, 2007) studied common mean of several log-normal populations.

Some comments under the section “The proposed methods for confidence interval estimation of common gamma mean” are added to reflect this point.

2. Line 113, Step 3. Is it Eq (8) or Eq (3)?

Response:

The Step 3 of computing algorithm on Page 5 line 119 (previous line 113) refers to Eq (12). The Eq (3) refers to pooled estimate of sample means. The Eq (8) refers to the generalized pivot of single Gamma mean, while Eq (12) refer to the generalized pivot of the common mean.

The common mean problem of the gamma distribution has been well identified in the literature, as the author claimed. There must be some existing methods in constructing the confidence interval. The author needs to compare the proposed methods with the existing ones using simulations.

Response:

Thanks for the comments.

While the common mean of gamma distribution is a meaningful research topic, there does not exit methods which can provide confidence interval with decent performance at finite sample sizes. In page 2, some large

sample approach was presented. However, as expected, its confidence intervals can be severely liberal. Hence the results were not presented in the paper.

To clarify this point, under “Simulations studies”, some comments are added.

4. For the examples, why is it important to assume a common mean for the two samples? It seems that confidence intervals can be well constructed for each individual sample.

Response:

Thanks for this insightful question.

When the comment mean assumption is satisfied, using all the sample to estimate the confidence intervals can lead to more efficient estimates, i.e. shorter confidence intervals. Hence it is important to utilize the assumption of common mean when it is satisfied.

Thank you for your comments.
