# Supplementary material for: Confidence interval estimation of the common mean of several gamma populations
Source: PLoS One. 2022 Jun 17;17(6):e0269971. doi: 10.1371/journal.pone.0269971 (PMC9205481; doi:10.1371/journal.pone.0269971)
Supplement: Attachment — Submitted filename: Responses to reviewer 2_R2.pdf [file pone.0269971.s003.pdf]

Response to Reviewer 2 (PONE-D-21-27089R1): "Confidence Interval Estimation of the Common Mean of Several Gamma Populationsg" by Yan

We appreciate your second round of reading and valuable comments/suggestions which greatly improved this article. For your consideration, the following point-to-point responses are provided.

Firstly, in my previous Comment#1, what I asked is whether the constructed GPQs are valid. There are two conditions for a valid GPQ and the author needs to check them. In addition, it is good to see the superb performance of the GPQ method, but is there any theoretical guarantee? If no, probably the good performance is just because of the simulation settings. The author needs to provide some insights in using GPQs.

Response:

Thanks for the questions/comments.

On page 5, two conditions for GPQ to be valid were added. Please note that the GPQ R_μ is an approximate bona fide generalized pivotal quantity because the GPQ for single gamma mean is approximate.

Our paper provides an approach for making inference about common gamma mean using generalized inference (fiducial inference). It is intended to be an applied paper to demonstrate the good performance of fiducial inference under one particular practical setting.

Hanig published a series of theoretical papers (e.g. Statistica Sinica, 2009; JASA 2016) which touched on theoretical justifica-

tions of fiducial intervals. These work are highly theoretical, but still shed lights on the general justification for making fiducial inference.

The impressive performance of generalized inference for a variety of practical problems have been demonstrated by many existing generalized inference papers using simulations studies. Our paper serves an addition in this category.

Secondly, in the response to my Comment#3, the authors mentioned all other methods do not perform well at finite sample sizes. I do not see the rational between the two sentences ""In page 2, some large sample approach was presented" and "However, as expected, its confidence intervals can be severely liberal". The authors clearly do not conduct numerical simulations so what is the evidence of "expectation"? I would suggest adding simulation results from other methods to the paper to better highlight the contribution.

Response:

Thanks for the suggestion.

In the following, a simulation table for the large sample approach in equation (4) in the paper is presented. We used the setting corresponding to all simulation scenarios in Table 1 and 2 in the paper. As you can see, the performances is generally too liberal, as expected. Hence the results are not presented in "Simulation studies". There is a sentence at end of "Simulation Studies" on page 7 to state this point.

Thanks for your comments.

Table S1: Simulation results for the large sample approach ($K = 2$)

K	μ	shape	Sizes	n	CP	CI
2	1	(0.5,1)	I	(5,5)	0.647	0.975
			II	(5,10)	0.703	0.845
			III	(10,10)	0.774	0.852
			IV	(20,20)	0.852	0.654
			V	(50,50)	0.912	0.438
		(1,2)	I	(5,5)	0.730	0.737
			II	(5,10)	0.764	0.635
			III	(10,10)	0.821	0.616
			IV	(20,20)	0.889	0.472
			V	(50,50)	0.908	0.309
		(1,10)	I	(5,5)	0.750	0.412
			II	(5,10)	0.835	0.340
			III	(10,10)	0.873	0.335
			IV	(20,20)	0.916	0.251
			V	(50,50)	0.936	0.163
		(2,10)	I	(5,5)	0.781	0.387
			II	(5,10)	0.840	0.323
			III	(10,10)	0.860	0.319
			IV	(20,20)	0.907	0.239
			V	(50,50)	0.938	0.157
		(5,10)	I	(5,5)	0.782	0.343
			II	(5,10)	0.839	0.299
			III	(10,10)	0.868	0.284
			IV	(20,20)	0.903	0.212
			V	(50,50)	0.937	0.139
	5	(0.5,1)	I	(5,5)	0.635	4.851
			II	(5,10)	0.712	4.316
			III	(10,10)	0.767	4.189
			IV	(20,20)	0.848	3.264
			V	(50,50)	0.908	2.168
		(1,2)	I	(5,5)	0.707	3.660
			II	(5,10)	0.758	3.175
			III	(10,10)	0.826	3.074
			IV	(20,20)	0.875	2.344
			V	(50,50)	0.929	1.554
		(1,10)	I	(5,5)	0.751	2.050
			II	(5,10)	0.840	1.691
			III	(10,10)	0.858	1.677
			IV	(20,20)	0.908	1.246
			V	(50,50)	0.930	0.822
		(2,10)	I	(5,5)	0.788	1.962
			II	(5,10)	0.838	1.621
			III	(10,10)	0.871	1.584
			IV	(20,20)	0.918	1.200
			V	(50,50)	0.933	0.784
		(5,10)	I	(5,5)	0.766	1.712
			II	(5,10)	0.839	1.489
			III	(10,10)	0.877	1.428
			IV	(20,20)	0.923	1.071
			V	(50,50)	0.931	0.696
5	1	(0.5,0.5,0.75,0.75,1)	VI	(5,5,5,5,5)	0.386	0.519
			VII	(10,10,10,10,10)	0.547	0.491
			VIII	(20,20,20,20,20)	0.717	0.404
			IX	(5,10,10,20,50)	0.641	0.371
			X	(50,50,50,50,50)	0.846	0.280
		(0.5,1,2,5,10)	VI	(5,5,5,5,5)	0.601	0.280
			VII	(10,10,10,10,10)	0.762	0.243
			VIII	(20,20,20,20,20)	0.856	0.188
			IX	(5,10,10,20,50)	0.830	0.147
			X	(50,50,50,50,50)	0.925	0.125
		(0.5,2,2,5,5)	VI	(5,5,5,5,5)	0.583	0.306
			VII	(10,10,10,10,10)	0.766	0.271
			VIII	(20,20,20,20,20)	0.869	0.212
			IX	(5,10,10,20,50)	0.805	0.183
			X	(50,50,50,50,50)	0.921	0.141
		(1,2,2,5,5)	VI	(5,5,5,5,5)	0.591	0.304
			VII	(10,10,10,10,10)	0.772	0.267
			VIII	(20,20,20,20,20)	0.865	0.208
			IX	(5,10,10,20,50)	0.833	0.183
			X	(50,50,50,50,50)	0.916	0.139
		(2,2,5,5,10)	VI	(5,5,5,5,5)	0.639	0.246
			VII	(10,10,10,10,10)	0.800	0.214
			VIII	(20,20,20,20,20)	0.882	0.166
			IX	(5,10,10,20,50)	0.861	0.141
			X	(50,50,50,50,50)	0.944	0.110
	5	(0.5,0.5,0.75,0.75,1)	VI	(5,5,5,5,5)	0.384	2.562
			VII	(10,10,10,10,10)	0.569	2.472
			VIII	(20,20,20,20,20)	0.713	2.028
			IX	(5,10,10,20,50)	0.643	1.844
			X	(50,50,50,50,50)	0.848	1.402
		(0.5,1,2,5,10)	VI	(5,5,5,5,5)	0.602	1.409
			VII	(10,10,10,10,10)	0.774	1.214
			VIII	(20,20,20,20,20)	0.870	0.945
			IX	(5,10,10,20,50)	0.838	0.736
			X	(50,50,50,50,50)	0.920	0.625
		(0.5,2,2,5,5)	VI	(5,5,5,5,5)	0.579	1.535
			VII	(10,10,10,10,10)	0.748	1.359
			VIII	(20,20,20,20,20)	0.847	1.056
			IX	(5,10,10,20,50)	0.809	0.916
			X	(50,50,50,50,50)	0.915	0.705
		(1,2,2,5,5)	VI	(5,5,5,5,5)	0.610	1.522
			VII	(10,10,10,10,10)	0.770	1.346
			VIII	(20,20,20,20,20)	0.864	1.043
			IX	(5,10,10,20,50)	0.827	0.910
			X	(50,50,50,50,50)	0.915	0.691
		(2,2,5,5,10)	VI	(5,5,5,5,5)	0.640	1.231
			VII	(10,10,10,10,10)	0.785	1.070
			VIII	(20,20,20,20,20)	0.877	0.826
			IX	(5,10,10,20,50)	0.858	0.706
			X	(50,50,50,50,50)	0.927	0.548
